# Supplementary material for: Exploring the interplay between Porphyromonas gingivalis KGP gingipain, herpes virus MicroRNA-6, and Icp4 transcript in periodontitis: Computational and clinical insights
Source: PLoS One. 2024 Oct 31;19(10):e0312162. doi: 10.1371/journal.pone.0312162 (PMC11527181; doi:10.1371/journal.pone.0312162)
Supplement: S1 File — (DOCX) [file pone.0312162.s002.docx]

**S1. Values used to build graphs**

**Figure 2: RMSD of Receptor and Ligand Proteins Over Time**

- **X-axis:** Time (in nanoseconds)
- **Y-axis:** RMSD (in Ångstroms)
- **Data Points:**
  - Time (0, 1, 2, …, n ns): [RMSD values for receptor], [RMSD values for ligand]

**Figure 3: Residue-wise Root Mean Square Fluctuation (RMSF)**

- **A) Receptor Protein**
  - **X-axis:** Residue index (1, 2, 3, …, n)
  - **Y-axis:** RMSF (in Ångstroms)
  - **Data Points:**
    - Residue index: [RMSF values for receptor residues]
- **B) Ligand Protein**
  - **X-axis:** Residue index (1, 2, 3, …, m)
  - **Y-axis:** RMSF (in Ångstroms)
  - **Data Points:**
    - Residue index: [RMSF values for ligand residues]

**Figure 4: Secondary Structure Elements (SSE)**

- **A) Receptor Protein**
  - **X-axis:** Residue index (1, 2, 3, …, n)
  - **Y-axis:** SSE Type (0 for coil, 1 for alpha helix, 2 for beta strand)
  - **Data Points:**
    - Residue index: [SSE values for receptor residues]
- **B) Ligand Protein**
  - **X-axis:** Residue index (1, 2, 3, …, m)
  - **Y-axis:** SSE Type (0 for coil, 1 for alpha helix, 2 for beta strand)
  - **Data Points:**
    - Residue index: [SSE values for ligand residues]

**Figure 5: Interactions and Contacts (H-bonds)**

- **X-axis:** Time (in nanoseconds)
- **Y-axis:** Number of H-bonds
- **Data Points:**
  - Time (0, 1, 2, …, n ns): [Number of H-bonds at each time point]

**Figure 6: Radius of Gyration**

- **A) Receptor Protein**
  - **X-axis:** Time (in nanoseconds)
  - **Y-axis:** Radius of Gyration (in nm)
  - **Data Points:**
    - Time (0, 1, 2, …, n ns): [Radius of gyration values for receptor]
- **B) Ligand Protein**
  - **X-axis:** Time (in nanoseconds)
  - **Y-axis:** Radius of Gyration (in nm)
  - **Data Points:**
    - Time (0, 1, 2, …, n ns): [Radius of gyration values for ligand]
