# Supplementary figures and images for: Exploring the interplay between Porphyromonas gingivalis KGP gingipain, herpes virus MicroRNA-6, and Icp4 transcript in periodontitis: Computational and clinical insights
Source: PLoS One. 2024 Oct 31;19(10):e0312162. doi: 10.1371/journal.pone.0312162 (PMC11527181; doi:10.1371/journal.pone.0312162)

S2\_raw\_images

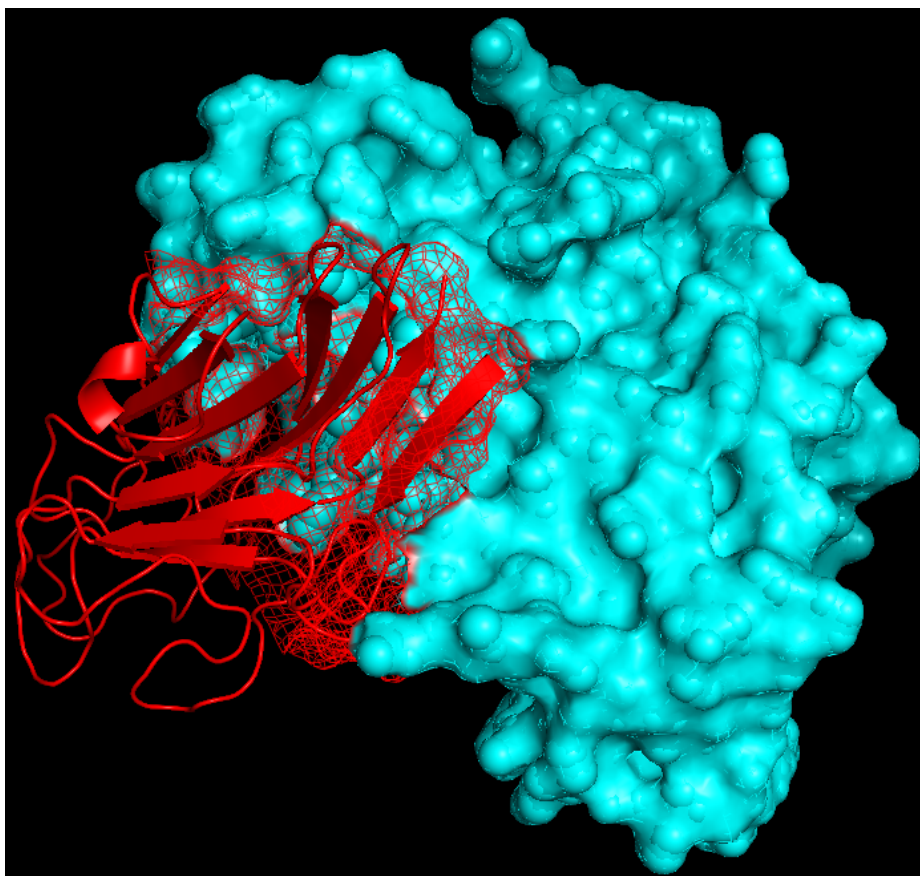

**Figure 1.**

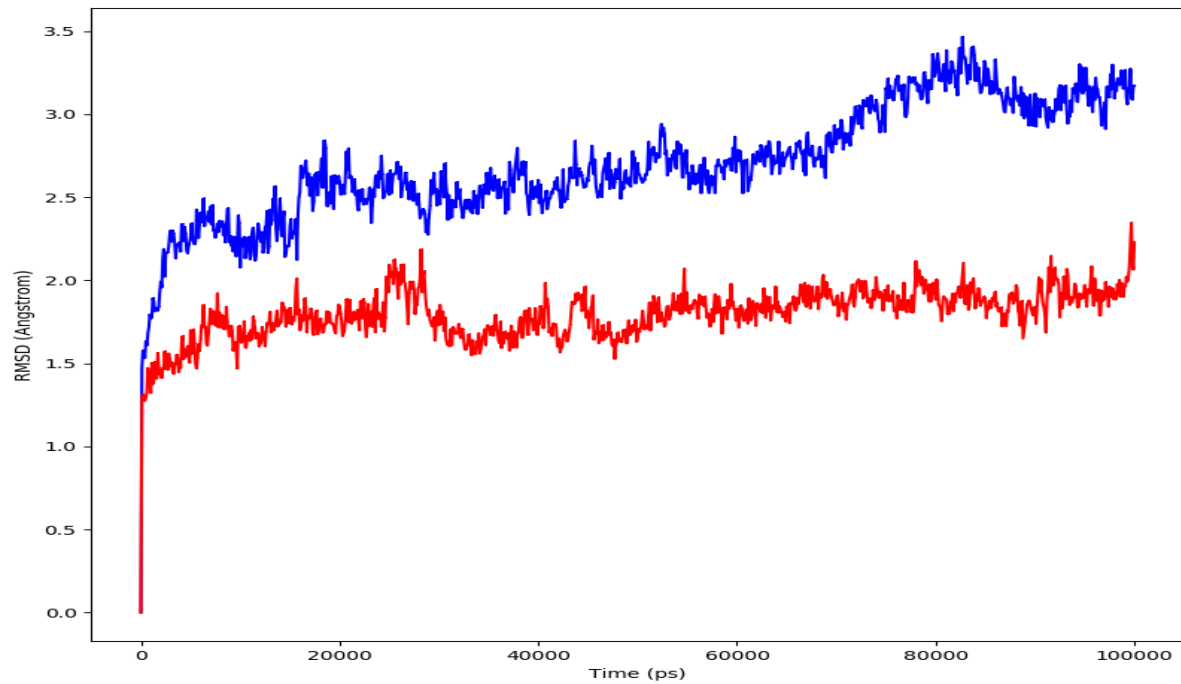

**Figure 2.**

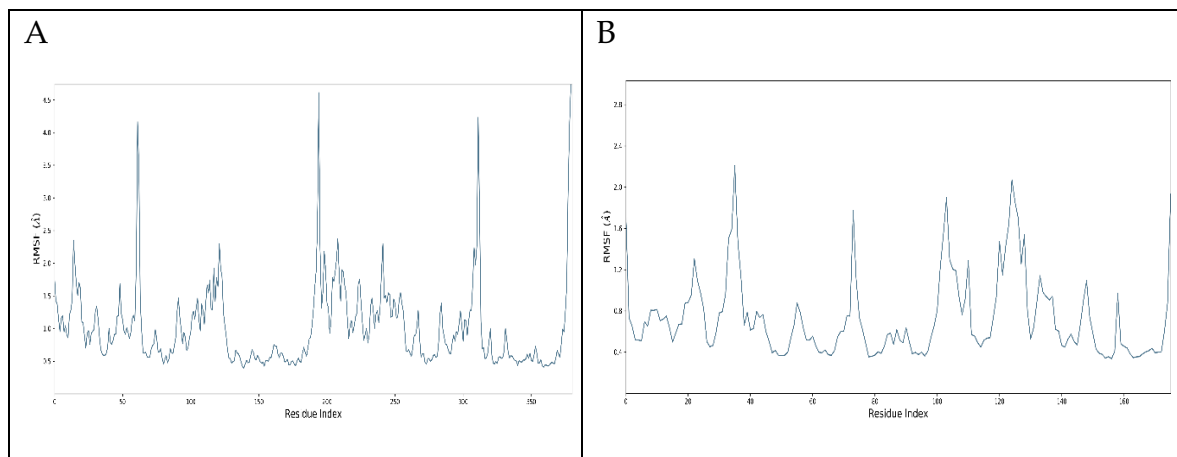

**Figure. 3**

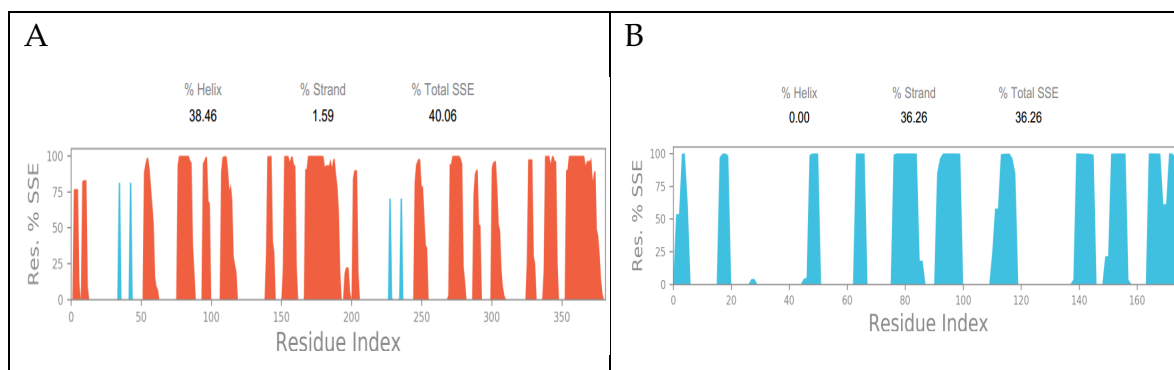

**Figure 4.**

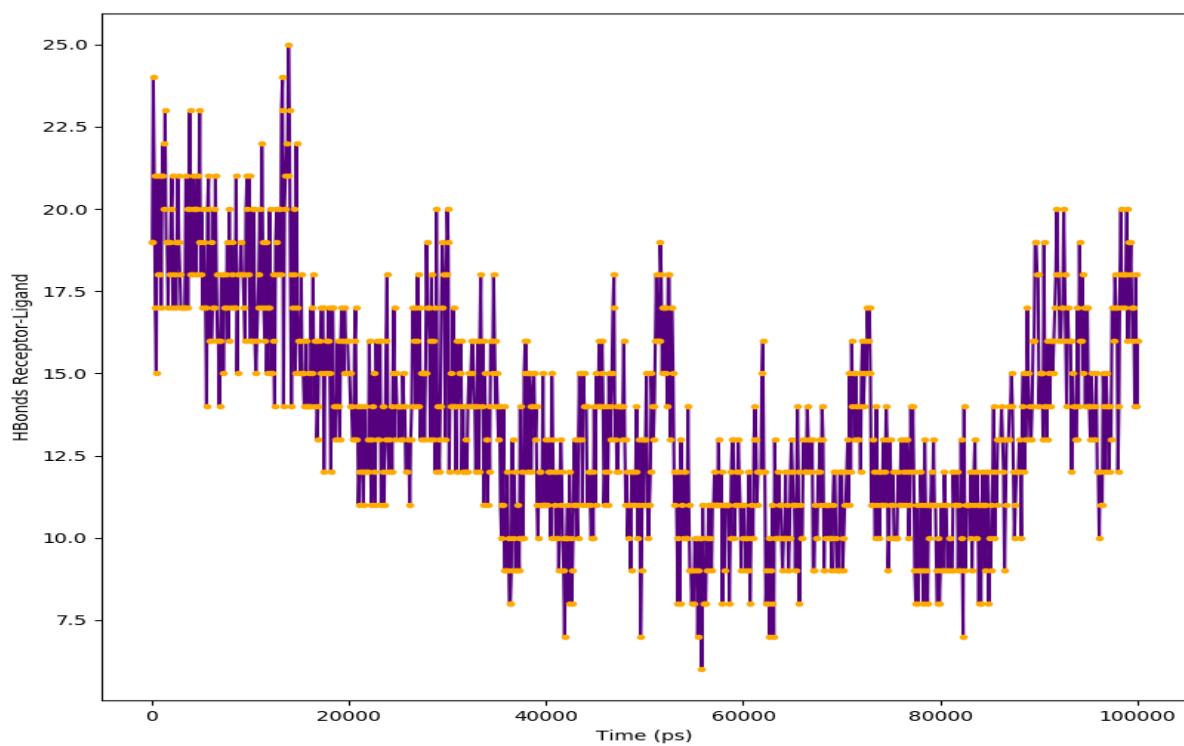

**Figure 5.**

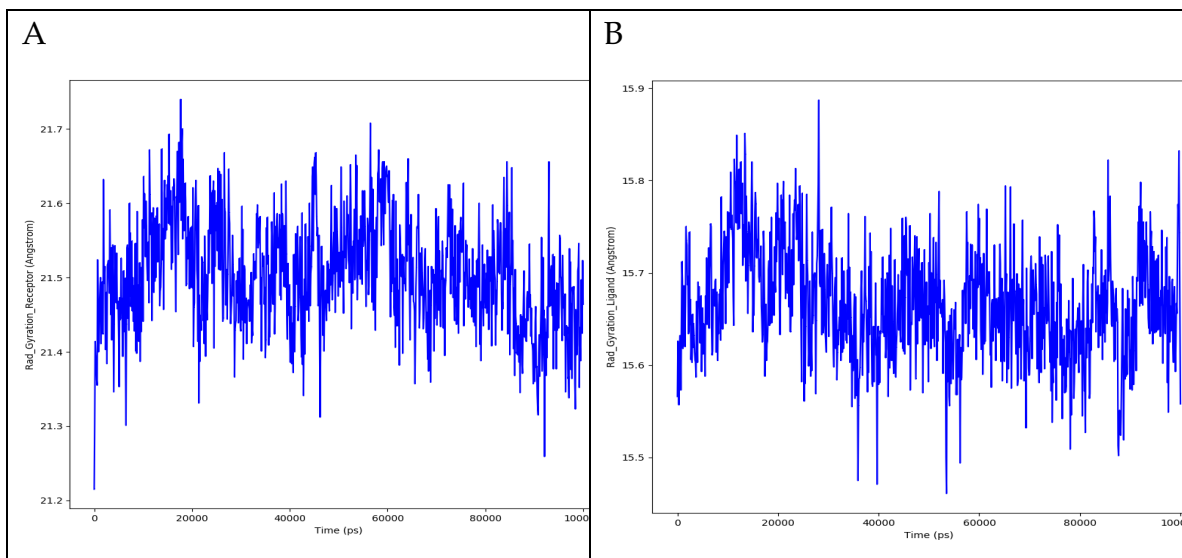

**Figure 6.**

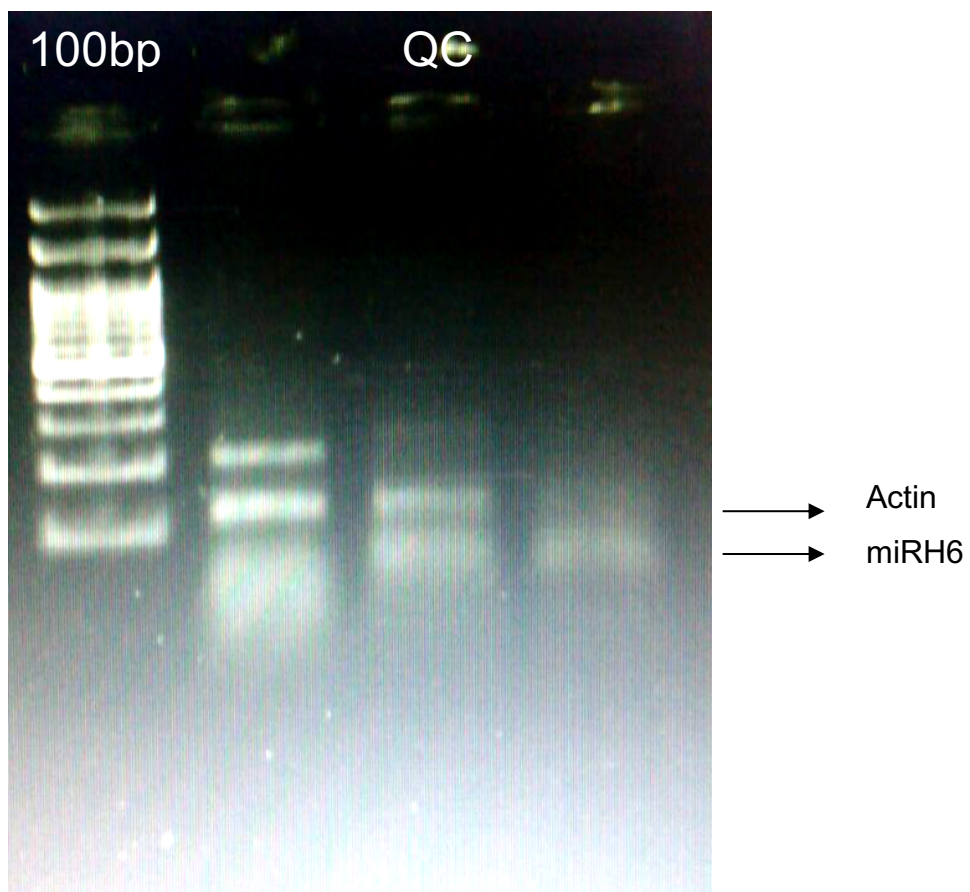

**Figure 7.**

Supplement: S1 Raw images — (PDF) [file pone.0312162.s003.pdf]
